# Congenital Myasthenic Syndrome-4C in a Consanguineous Romani Family: Genetic Insights and Clinical Implications

**DOI:** 10.3390/diagnostics15030235

**Published:** 2025-01-21

**Authors:** Codruta Diana Petchesi, Aurora Alexandra Jurca, Alexandru Daniel Jurca, Florica Ramona Dorobantu, Alin Remus Iuhas, Emilia Severin, Claudia Maria Jurca

**Affiliations:** 1 Department of Preclinical Disciplines, Faculty of Medicine and Pharmacy, University of Oradea, 1 December Sq., 410081 Oradea, Romania; alexjurca@uoradea.ro (A.D.J.); claudiajurca@uoradea.ro (C.M.J.); 2 Regional Center of Medical Genetics Bihor, County Emergency Clinical Hospital Oradea (Part of ERN ITHACA), 410053 Oradea, Romania; 3 Doctoral School of Biomedical Sciences, University of Oradea, 410087 Oradea, Romania; jurca.auroraalexandra@student.uoradea.ro; 4 Department of Clinical Disciplines, Faculty of Medicine and Pharmacy, University of Oradea, 410081 Oradea, Romania; rdorobantu@uoradea.ro (F.R.D.); alin.iuhas@yahoo.com (A.R.I.); 5 County Emergency Clinical Hospital Oradea, 410167 Oradea, Romania; 6 Department of Genetics, “Carol Davila” University of Medicine and Pharmacy–Bucharest, Dionisie Lupu Street, Number 37, District 2, 020021 Bucharest, Romania

**Keywords:** congenital myasthenic syndrome, *CHRNE* gene, co-sanguinity

## Abstract

**Background and Clinical Significance**: Congenital myasthenic syndrome-4C (CMS4C) associated with acetylcholine receptor (AChR) deficiency is an autosomal recessive defect of the motor endplate caused by homozygous or compound heterozygous mutations in the *CHRNE* gene on chromosome 17p13. **Case Presentation**: The authors present a familial case of CMS4C with three affected children in a consanguineous Romani family. Muscle weakness, fatigue, and ocular muscle impairment were present in all cases; two of the three siblings had delayed motor milestones, highly arched palates, and facial weakness. None of the children expressed bulbar symptoms. One child expressed a severe form, with recurrent respiratory infections, and multiple hospitalizations, while the other siblings expressed a mild phenotype, without hospital admissions. Repetitive nerve stimulation showed a myasthenic-type decrement greater than 10% of several muscles. A pathogenic frameshift variant (NM_000080.4: c.1327del) in the *CHRNE* gene was found in a homozygous status in all the affected children and in both parents. After 6 months of Pyridostigmine and Salbutamol treatment, the evolution of the case was good, with the improvement of most of the signs and no need for hospitalization. **Conclusions**: Early genetic diagnosis and appropriate therapy in the context of a multidisciplinary approach is mandatory for an optimal long-term prognosis. Community-wide carrier screening through comprehensive genetic testing is imperative to ensure accurate genetic counseling in genetic isolates. The authors report this case due to the increased number of affected children in a consanguine family from a small Romani community.

## 1. Introduction

Congenital myasthenic syndromes (CMSs) are a group of extremely rare genetic disorders of the neuromuscular junction, characterized by muscle weakness [1]. The frequency of CMSs is unknown, with around 600 affected families having been described [2]. CMSs exhibit genetic heterogeneity, with mutations in 35 genes coding for presynaptic, synaptic, and postsynaptic proteins involved in neuromuscular transmission having been detected [3]. Based on the location of the affected protein within the neuromuscular junction (presynaptic, synaptic, or postsynaptic), several CMS subtypes are recognized.

Congenital myasthenic syndrome-4C (CMS4C) associated with acetylcholine receptor (AChR) deficiency is an autosomal recessive defect of the motor endplate caused by homozygous or compound heterozygous mutations in the *CHRNE* gene on chromosome 17p13 [4,5,6].

AChRs are pentameric protein complexes that mediate signal transmission at the neuromuscular junction [7]. There are two isoforms of AchR, embryonic and adult acetylcholine receptors, which differ in subunit composition, physiological properties, and functional roles at the neuromuscular junction during development and maturity. The embryonic form typically has a subunit composition of α2βγδ, where the gamma (γ) subunit is present. In adult AChRs, the gamma (γ) subunit is replaced by an epsilon (ε) subunit, giving them a composition of α2βεδ. This substitution, which usually occurs during the first two weeks after birth, shortens the ion channel’s open time, leading to faster and more precise signaling [8,9,10]. Such rapid responses are essential for efficient and coordinated muscle contractions in mature muscle fibers.

The *CHRNE* gene (#100725) is located on chromosome 17 (17p13.2) and contains 12 exons encoding the ε-subunit of the Acetylcholine receptor [11,12]. Mutations in CHRNE gene can disrupt the ε-subunit structure and function; this disruption impairs cell-to-cell signaling and results in muscle weakness, delayed motor skill development, and reduced skeletal muscle movement [13,14,15].

Clinical onset typically occurs at birth or in early childhood, though it can rarely present later in life [16]. Characteristic features include extreme fatigue and weakness affecting various muscle groups, such as the ocular muscles, bulbar muscles, and limb muscles [17]. Clinical manifestations vary among families; while some individuals present only ocular symptoms, others develop generalized myasthenia, and in severe cases, respiratory difficulties may occur [18,19].

Treatment with acetylcholinesterase inhibitors can improve neuromuscular transmission. Following the administration of salbutamol and pyridostigmine, approximately 80% of symptoms resolve [20].

## 2. Case Presentation

### 2.1. Methods

Written informed consent was obtained from the parents prior to study participation for genetic testing, the unprocessed presentation of photographs, and publication.

**Figure 1:**
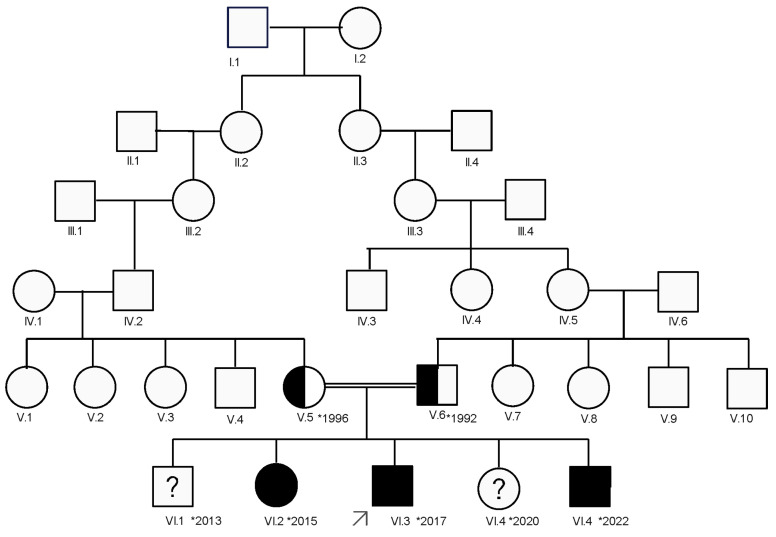
The family tree: a six-generation Romani family with three affected children (VI.2, VI.3, and VI.5). The index case is the third child in the last generation, marked by an arrow.

The index case (VI.3) is a 4-year-old boy who was referred for genetic evaluation by a pediatric neurologist in 2021 due to muscle weakness and fatigability. He is the third child of a consanguineous Romani couple who are third-degree cousins. Following a detailed family history, a pedigree was constructed using an in-house tool (Figure 1), which revealed another affected sibling (VI.2), a 6-year-old girl previously known to pediatric neurology for hypotonia and muscle weakness.

After the family moved abroad and missed two years of follow-up, they returned with a third affected child (VI.5), who presented with a similar clinical phenotype (Figure 1).

Subsequent to the physical examination, a comprehensive laboratory evaluation was conducted to assess the children’s hematologic, metabolic, and endocrine parameters. This included a complete blood count to identify potential anemia or infection, serum creatine kinase (CK-MM) to detect muscle injury, hepatic and renal function tests to evaluate organ integrity, thyroid hormone assays to rule out endocrine dysfunction, and blood glucose measurements to screen for glycemic abnormalities. Electromyography (EMG) and nerve conduction studies (NCS) completed the neurological examination.

Peripheral blood samples were collected from the three affected children (VI.2, VI.3, and VI.5) and their parents (V.5 and V.6). All molecular investigations were conducted at the Regional Center of Medical Genetics Dolj, Romania. DNA was extracted using standard protocols. Exome sequencing was performed with the TruSeq Exome Panel on an Illumina NetSeq 550 IVD platform. Library preparation was carried out using the Illumina DNA Prep with Enrichment Kit. Data analysis utilized an in-house bioinformatics pipeline and was cross-referenced with established databases (OMIM, ClinVar, ORPHA, Varsome, Franklin, PubMed). Variants were interpreted according to the American College of Medical Genetics and Genomics (ACMG) guidelines, which classify variants as benign; likely benign; variant of unknown significance (VUS); likely pathogenic–pathogenic.

### 2.2. Clinical Evaluation of the Patient

The first patient, the index case (VI.3), was the third child of healthy, consanguineous parents. He was delivered spontaneously at 32 weeks of gestation in cephalic presentation, with Apgar scores of 7 at one minute and 9 at five minutes. Upon initial examination, he exhibited mild acrocyanosis, spontaneous movements with symmetrical limb activity, reduced flexor tone, and less vigorous root, suck, and grasp reflexes, alongside mild respiratory effort due to perinatal asphyxia. Transfontanellar ultrasonography showed mild ventricular asymmetry without bleeding, and neurological monitoring showed no signs of hypoxic–ischemic encephalopathy.

Subsequently, he exhibited mild difficulties in extrauterine life, specific to preterm newborns, and received care in the neonatal intensive care unit, requiring supplemental oxygen during the initial days, thermoregulatory support, and assisted feeding. Routine sepsis screenings and blood gas analyses were performed and yielded results within normal limits. He was discharged after 28 days, weighing 2550 g and demonstrating a coordinated suck–swallow–breathe reflex.

Over the course of his development, he continued to display mild generalized hypotonia and showed delayed motor milestones. He achieved head control by five months of age, sat without support at 12 months, and was able to walk independently by two years. He experienced frequent falls and fatigue during physical activities, which worsened following intense physical effort and later in the day. Additionally, he had recurrent bronchitis and bronchopneumonias, more than six episodes per year, requiring frequent hospitalizations (at least three per year), with symptomatic relief following Salbutamol therapy.

**Figure 2:**
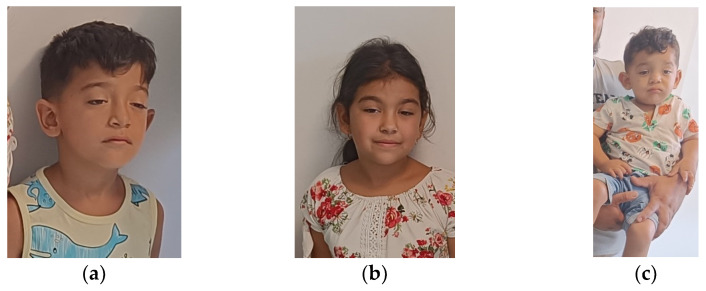
Clinical features of the three affected children: (**a**) index case, VI.3 (**b**) older sister, case VI.2; (**c**) younger sibling, case VI.5.

At the age of four, he was referred to a pediatric neurologist who recommended a genetic evaluation. His facial features included dolichocephaly, a narrow forehead, hypertelorism, bilateral ptosis, epicanthal folds, a broad nasal bridge, downward-turned corners of the mouth, multiple dental anomalies, and a small chin (Figure 2a). He also presented with pectus excavatum and generalized muscle weakness, accompanied by increased fatigability and frequent falls while walking.

The second affected individual in the family (VI.2) was a 6-year-old girl, the second child, born at term with a birth weight of 3300 g. Her facial characteristics included dolichocephaly, a narrow forehead, hypertelorism, bilateral ptosis, epicanthal folds, a broad nasal bridge, a highly arched palate, multiple dental anomalies, and a small chin (Figure 2b). She also presented with pectus excavatum.

Clinically, she suffered from asthma, frequently complicated by recurrent asthmatic bronchitis, which improved with outpatient treatment and did not require hospitalization. Additionally, she experienced fatigability, muscle weakness, and frequent falls while walking. Although her parents noticed certain signs during the first months of life, they did not seek specialist consultation at the time since her motor development was not significantly delayed.

Following the initial evaluation, contact with the family was lost when they moved abroad for two years. In 2023, they returned, and the index case required hospitalization in the intensive care unit for respiratory failure. During a subsequent genetic re-evaluation, a third affected child in the family (VI.5) was identified.

This individual, the fifth child of the family, was born at term after normal in-utero movements, with a birth weight of 3900 g. He presented with a similar facial appearance, including dolichocephaly, a narrow forehead, hypertelorism, bilateral ptosis, dystopia canthorum, a broad nasal bridge, protruding ears, facial weakness, a highly arched palate, multiple dental anomalies, and a small chin (Figure 2c). Hepatosplenomegaly was noted prenatally, and postnatal motor development was delayed (sat without support at 11 months, independent walking was not acquired at 18 months) (Table 1).

Genetic testing was carried out, and the index case was started on pyridostigmine therapy, resulting in significant improvement across all muscle groups.

### 2.3. Laboratory Investigations

In all patients, complete blood count, hepatic and renal function assessments, thyroid hormone levels, and blood glucose measurements were within standard reference intervals. Serum CK-MM also remained within normal limits (in the last evaluation, 105 U/L for the index case, respectively, 102 U/L for case VI.2 and 96 U/L for case VI.5), and assays for anti-acetylcholine receptor (anti-AChR) and anti-muscle-specific kinase (anti-MuSK) antibodies were negative.

### 2.4. Electromyography

Electromyographic and nerve conduction studies were performed for the index case (VI.3) and the second case (VI.2) using low-frequency (3 Hz) repetitive nerve stimulation (RNS). Electromyographic and nerve conduction studies were not performed in the third case due to financial constraints.

In the index case, who was receiving pyridostigmine treatment, distal latencies, compound muscle action potential (CMAP or M-wave) amplitudes, and motor conduction velocities were normal in the median, ulnar, tibial, and peroneal nerves. Sensory nerve action potential (SNAP) amplitudes and sensory conduction velocities were also normal when examining the median, ulnar, radial, sural, and superficial peroneal nerves, thereby excluding mononeuropathy or peripheral polyneuropathy.

**Figure 3:**
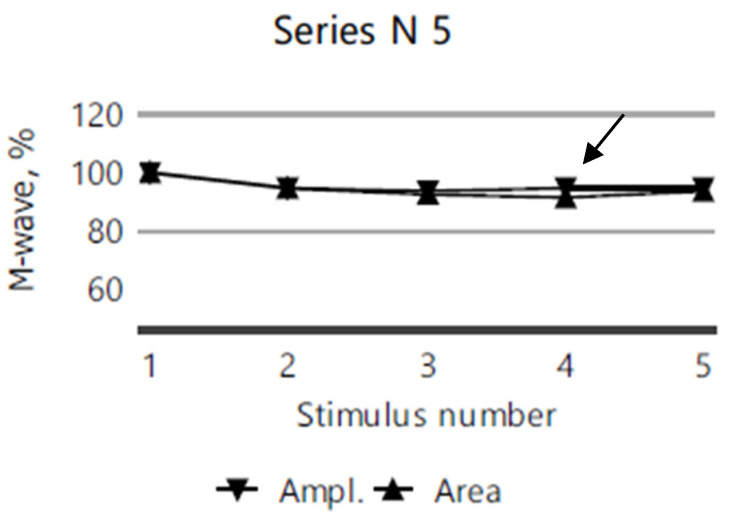
A 12% decrease in M-wave amplitude and area after the third repetitive nerve stimulation of the right orbicularis oculi in the 5th series of RNS for the index case.

**Figure 4:**
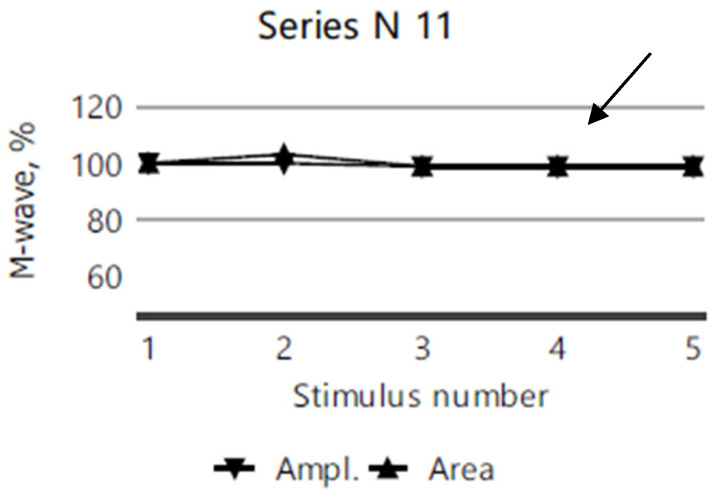
No decrement in the M-wave amplitude or area following five repeated nerve stimulations of the right accessory nerve in the 11th RNS series for the index case.

The repetitive nerve stimulation (11 series, each with 5 stimuli) of the bilateral facial nerves (recorded from the orbicularis oculi muscle), the right ulnar nerve (recorded from the abductor digiti minimi muscle), and the right accessory nerve (recorded from the superior portion of the trapezius muscle) revealed a myasthenic-type decrement in the right orbicularis oculi. In more than half of the series, M-wave amplitude and area decrement values of 12–13–15% were observed after the third repetitive nerve stimulation compared to the first stimulation (Figure 3), while no clear decrement compared to the first stimulation, was noted in any other muscle groups tested (Figure 4).

In the second case, who had not yet received treatment at the time of examination, 10 series of RNS, each with 5 stimuli, were performed. Distal latencies, M-wave amplitudes, and motor conduction velocities were normal for the median, ulnar, tibial, and peroneal nerves. SNAP amplitudes and sensory conduction velocities were also normal for the median, ulnar, radial, sural, and superficial peroneal nerves, thus excluding mononeuropathy or peripheral polyneuropathy.

**Figure 5:**
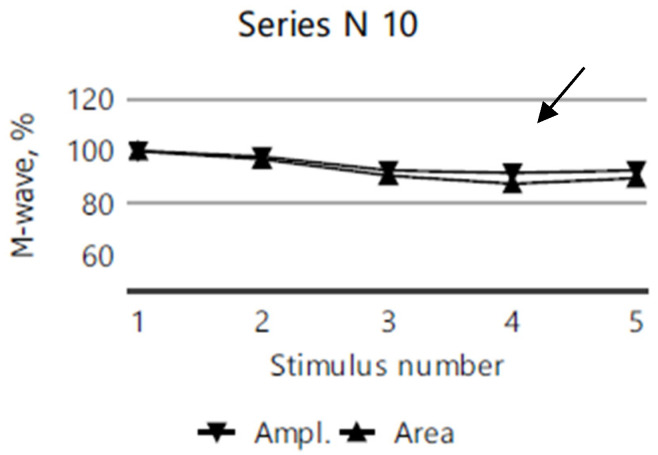
A more than 10% decrease in the M-wave amplitude and area after the third stimulation of the right ulnar nerve in the 10th RNS series for case VI.2.

**Figure 6:**
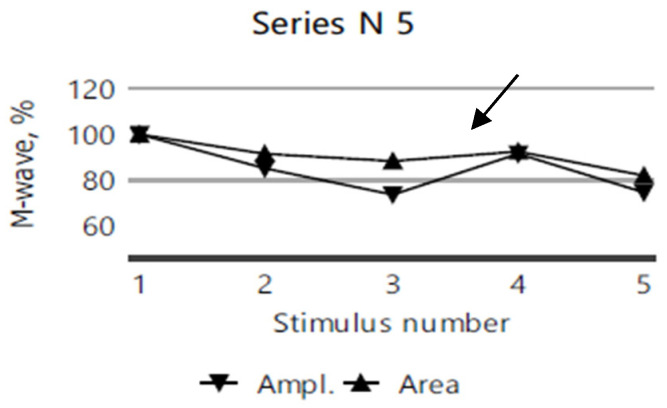
A more than 20% decrease in the M-wave amplitude and area after the third stimulation of the right facial nerve in the 5th RNS series for case VI.2.

**Figure 7:**
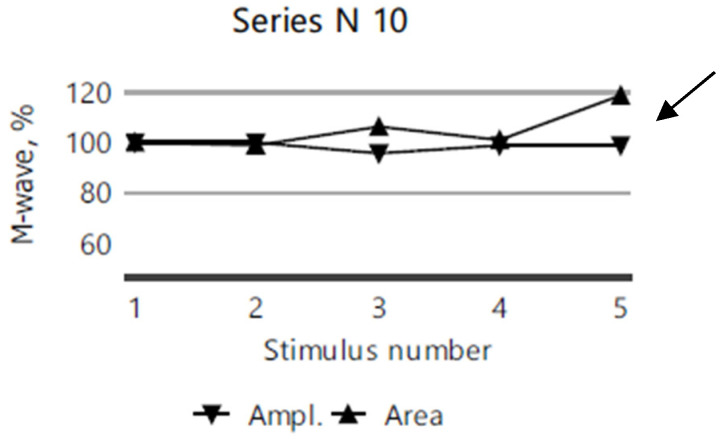
No M-wave amplitude or area decrement in the 10th RNS series of the right accessory nerve stimulation for case VI.2.

After the third stimulation, RNS revealed a myasthenic-type amplitude and area decrement (M-wave) compared to the first stimulation, in the right ulnar nerve (recorded from the abductor digiti minimi muscle; Figure 5) and in the facial nerve (recorded from the orbicularis oculi muscle; Figure 6). However, the repetitive nerve stimulation of the right accessory nerve (recorded from the superior portion of the trapezius muscle) showed no decrement compared to the first stimulation (Figure 7).

### 2.5. Molecular Investigations

A pathogenic frameshift variant (NM_000080.4: c.1327del) in the *CHRNE* gene was found in a homozygous status in all the affected children, and in heterozygous status in both parents. Because of financial constraints, further genetic testing was not conducted for other family members.

## 3. Discussion

### 3.1. Gene and Genetics

The pathogenic *CHRNE* gene variant identified in our case is a frameshift mutation in exon 12, encoding for the C-terminal region of the epsilon subunit. This region is crucial for proper receptor assembly, stability, and interaction with other subunits. It also contributes to the formation and stabilization of the receptor’s transmembrane domain, which is essential for the channel’s ion conduction. By altering the reading frame, the deletion disrupts the protein’s structure, resulting in a non-functional epsilon subunit. Consequently, the acetylcholine receptor cannot assemble or function properly, ultimately leading to muscle weakness and other characteristic symptoms.

The variant is classified as pathogenic (Class 5) based on the combination of the following ACMG/AMP criteria, which provide compelling support for the pathogenic nature of this frameshift variant:

PVS1 (pathogenic very strong): This variant is a null variant (frameshift) predicted to cause loss of function in CHRNE, consistent with a well-established loss-of-function disease mechanism for congenital myasthenic syndrome.

PM2 (pathogenic moderate): This variant is extremely rare or absent in large population databases (e.g., gnomAD), indicating that it is unlikely to be a benign polymorphism.

PP1 (supporting evidence): Family-based segregation analysis indicates that all affected children are homozygous for the c.1327del variant, while both parents are heterozygous and unaffected, reflecting autosomal recessive inheritance and further supporting pathogenicity.

PP3 (supporting evidence): Bioinformatics predictions suggest a severe impact on the protein.

The c.1327del mutation in the *CHRNE* gene was initially reported as pathogenic in 2016. More recently, in 2024, Kastreva et al. identified 91 Bulgarian Romani patients carrying this pathogenic variant [21]. Despite the genetic homogeneity, the affected individuals demonstrated significant phenotypic heterogeneity.

The Romani population is believed to have originated from a small group of founders who migrated from northern India to Europe around 1000 years ago, leading to the increased frequency of certain genetic mutations within the population (founder mutations). One such mutation, 1267delG in the *CHRNE* gene, causes CMS4C and appears to be found exclusively in the Romani population, as well as in individuals of Indian and Pakistani ancestry [22,23,24].

Consanguinity and the founder effect have also been described by Richard et al., who reported a homozygous 1-bp insertion in the *CHRNE* gene in 14 families [25]. The identified pathogenic variant is associated with a mild, stable disease course, and the founder event from which it arose is estimated to have occurred at least 700 years ago. In Spain, *CHRNE* mutations, including several founder variants, are the most common cause of CMS, accounting for 27% of all CMS cases [26].

Our patients belong to a large family (Figure 1), with no other affected members reported in the first five generations. They are part of a small Romani community characterized by limited genetic mixing with other populations and a relatively high prevalence of consanguinity. In this particular family, the parents are third-degree cousins, both carriers of the mutation. Within this close-knit community (around 200 individuals), another CMS4C case caused by the same pathogenic variant has also been identified a few years ago.

### 3.2. Clinical and Paraclinical Aspects

Currently, there are no definitive diagnostic criteria for congenital myasthenic syndromes. However, CMS is generally suspected under the following conditions: early-onset fatigable muscle weakness; a positive family history or a history suggestive of neonatal hypotonia; clinical and neurophysiological findings consistent with myasthenia in the absence of myasthenia-associated antibodies; electromyographic (EMG) evidence of a decremental response of 10% or more in the amplitude or area of the first compound muscle action potential (CMAP); single-fiber EMG findings indicative of neuromuscular junction dysfunction; and the presence of a specific clinical syndromic phenotype [27].

In our case, suspicion arose due to early-onset fatigable muscle weakness (primarily affecting the ocular, bulbar, and proximal limb muscles), clinical and neurophysiological features consistent with myasthenia, and the absence of antibodies. The identification of another affected individual within the same small community prompted us to test for CMS.

The clinical onset of CMS may occur as early as the neonatal period—or even prenatally—depending on the specific mutation involved [28,29]. In our family, the first signs appeared at birth for one child, and within the first few months of life for the other two siblings.

There is notable variability in clinical presentation, and the syndrome may sometimes overlap with seronegative myasthenia gravis [30].

Muscle weakness, fatigue, and ocular muscle impairment were observed in all three siblings. Two of them exhibited delayed motor milestones, a highly arched palate, and facial weakness. None of the children showed bulbar symptoms. One sibling presented with a severe phenotype, characterized by neonatal hypotonia, recurrent respiratory infections, and multiple hospitalizations, while the other siblings expressed a milder phenotype, without the need for hospital admission. Such phenotypic heterogeneity has been documented previously; Salih et al. (2011) described a similar family [31].

### 3.3. Treatment

Treatment with pyridostigmine at a dosage of 7 mg/kg/day was initiated in the patient with the severe phenotype (the index case). After six months of pyridostigmine and salbutamol therapy, the patient showed substantial improvement, with no further need for hospitalization. Electromyographic and conduction parameters normalized, except for persistent decrements in the orbicularis oculi, consistent with myopathic changes. Pyridostigmine treatment typically improves proximal muscle weakness, while ophthalmoparesis often remains unchanged, as demonstrated by Barisic in a 2020 study [32].

Over time, the other two siblings experienced progressive deterioration and subsequently began pyridostigmine therapy. They also showed clinical improvement within one month of treatment initiation.

As an alternative to acetylcholinesterase inhibitors, the potassium channel blocker 3,4-diaminopyridine (3,4-DAP) can be considered [33]. In 2023, Batheja et al. reported the case of a 4-year-old boy with a suboptimal response to pyridostigmine and albuterol who benefited from 3,4-DAP treatment [34].

### 3.4. Evolution and Monitoring

The natural history of congenital myasthenic syndromes (CMSs), particularly in adults, is not well characterized, and much of the available information comes from retrospective studies [35]. Early genetic diagnosis and the prompt initiation of therapy are crucial for achieving optimal outcomes [36]. It is recommended that muscle strength and respiratory function is regularly monitored, typically every six months in children and every twelve months in adults [20]. Our patients continue to undergo neurological assessment and treatment adjustments.

Although CMS typically follows a relatively benign course, clinical deterioration can occur during puberty and/or pregnancy [37]. Data regarding pregnancy in CMS remain limited; while pregnancy frequently leads to clinical exacerbations, most patients return to their pre-pregnancy clinical status within six months postpartum [38].

### 3.5. Genetic Counseling

Congenital myasthenic syndrome 4C is a monogenic disorder with an autosomal recessive inheritance pattern. In families where both parents are carriers, each child has a 25% risk of being affected. Each of the two untested children in the family has a 50% chance of being a carrier and a 25% chance of being healthy (non-carrier). All the offspring of affected individuals will be carriers. Given the family’s Romani background and the common practice of endogamy within this community, extended family testing is advisable to provide comprehensive genetic counseling.

## 4. Conclusions

CMS4C exhibits phenotypic heterogeneity, and most cases demonstrate a benign disease course. The case presented herein is noteworthy due to the unusually high number of affected children within a consanguineous family from a small Romani community, highlighting an elevated carrier frequency (four cases among approximately 200 individuals). Community-wide carrier screening through comprehensive genetic testing is imperative to ensure accurate genetic counseling in genetic isolates. Early genetic diagnosis and the timely initiation of appropriate therapy within a multidisciplinary care framework are essential for achieving an optimal long-term prognosis.

## Figures and Tables

**Table 1:** The main clinical features observed in the affected siblings.

Clinical Picture	Index Case (VI.3)	Older Sibling (VI.2)	Younger Sibling (VI.5).
Onset	At birth	Early age	Early age
Muscle fatigue	Present	Present	Present
Muscular weakness	Present	Present	Present
Bulbar symptoms	Not present	Not present	Not present
Ocular muscle impairment	Ptosis	Ptosis	Ptosis
Facial weakness	Present	Not present	Present
High-arched palate	Not present	Present	Present
Multiple dental anomalies	Present	Present	Present
Delayed motor milestones	Present	Not present	Present
Recurent respiratory infections: bronchitis and bronchopneumonias	Present	Not present	Not present
Pectus excavatum	Present	Present	Not present
Improvement with pyridostigmine	Present	Present	present

## Data Availability

The original contributions presented in this study are included in the article. Further inquiries can be directed to the corresponding authors.
